# Прогностические исследования в эндокринологии: дизайн и типичные ошибки

**DOI:** 10.14341/probl13702

**Published:** 2026-09-08

**Authors:** В. А. Аксёнов, О. Ю. Реброва

**Affiliations:** Российский национальный исследовательский медицинский университет им. Н.И. Пирогова Россия; Pirogov Russian National Research Medical University Russian Federation; Национальный медицинский исследовательский центр эндокринологии им. академика И.И. Дедова Россия; Endocrinology Research Centre Russian Federation

**Keywords:** дизайн исследования, когортные исследования, прогностическое исследование, анализ данных, методологические стандарты, study design, cohort studies, prognostic research, data analysis, methodology standards

## Abstract

Данная статья представляет собой аналитический обзор методологии и дизайна прогностических исследований. Оптимальным дизайном для прогностических исследований являются когортные исследования. В обзоре подробно рассматриваются различия между традиционными этиологическими когортными исследованиями и исследованиями, ориентированными на прогнозирование исходов. Приводятся примеры прогностических исследований, посвященных оценке риска гестационного сахарного диабета (ГСД), почечной недостаточности при сахарном диабете 2 типа (СД2), остеопоротических переломов у женщин в постменопаузе, результатов лечения бесплодия у женщин с повышенным уровнем фолликулостимулирующего гормона и выживаемости у пациентов с адренокортикальным раком. Подчеркивается важность соблюдения методологических стандартов, изложенных в руководствах STROBE и TRIPOD, а также необходимость предотвращения типичных методологических ошибок, таких как измерение предикторов после наступления исхода или использование ROC-анализа без предварительного построения прогностической модели. Особое внимание уделяется применению корректных статистических методов, поскольку их недостаточное понимание может привести к искаженным выводам и снижению научной достоверности результатов. Развитие прогностических когортных исследований и внедрение строгих аналитических подходов способствует повышению качества клинической практики и укреплению доказательной базы современной эндокринологии.

## Abstract (en)

This article provides a conceptual overview of the methodology and design of prognostic research. It presents cohort studies as the most suitable design for prognostic investigations and discusses in detail the distinctions between traditional etio logical cohort studies and those primarily aimed at predicting outcomes. The article reviews examples of prognostic studies that focus on predicting gestational diabetes mellitus, renal failure in type 2 diabetes mellitus, osteoporotic fractures in postmenopausal women, outcomes of infertility treatment in women with elevated follicle-stimulating hormone levels, and survival in patients with adrenocortical carcinoma. The importance of adhering to the methodological standards outlined in the STROBE and TRIPOD guidelines is emphasized. It also highlights the need to avoid common design and analytical pitfalls, such as measuring predictors after the outcome has occurred or using ROC analysis without developing a multivari able prediction model. Particular attention is given to employing appropriate statistical methods, as a lack of understanding of these methods may lead to biased inferences and diminish the scientific value of the published research. Incorporating well-designed prognostic cohort studies and rigorous analytical approaches will enhance clinical practice and strengthen the evidence base in modern endocrinology.

В современной медицине, включая эндокринологию, все большее внимание уделяется внедрению риск-ориентированных и персонализированных подходов к профилактике и лечению. Эти подходы предусматривают адаптацию медицинских вмешательств с учетом индивидуального уровня риска, то есть вероятности наступления определенного события у конкретного пациента в заданный период времени. Основная цель риск-ориентированной клинической практики состоит в создании индивидуализированных стратегий ведения пациентов и оптимизации планирования медицинской помощи, что позволяет существенно повысить ее результативность.

Прогностические модели разрабатываются и используются для оценки вероятности возникновения определенных событий у разных групп людей: тех, кто находится в группах риска, пациентов с установленными диагнозами, а также у бессимптомных индивидуумов. Такие модели применяются для прогнозирования вероятности рецидивов, появления осложнений или наступления летального исхода в рамках определенного временного периода после установления конкретного диагноза. Аналогично они могут применяться для предсказания исхода в течение определенного периода у лиц без установленного диагноза, например, для оценки вероятности развития сахарного диабета 2 типа (СД2) или остеопоротических переломов шейки бедра у лиц без остеопороза. В каждом из описанных сценариев количественная оценка прогноза помогает пациентам и клиницистам совместно решать, насколько оправдано проведение вмешательства. Если прогноз благоприятен и предполагает естественное улучшение состояния без вмешательства, то, скорее всего, в нем нет необходимости. С другой стороны, если прогноз остается неблагоприятным независимо от того, проводилось вмешательство или нет, целесообразность его проведения ставится под сомнение, учитывая связанные с ним риски и стресс, который оно может вызвать у пациента.

Историческим примером применения прогностических моделей в эндокринологии можно назвать прогностический индекс рака щитовидной железы EORTC, разработанный Баяром и его коллегами в 1979 г. [1]. Являясь одной из первых многофакторных моделей стратификации риска при раке щитовидной железы, индекс EORTC сыграл значимую историческую роль в развитии прогностических систем. Однако в современной клинической практике он уступил место более точным и проверенным прогностическим моделям.

## ОСОБЕННОСТИ ДИЗАЙНА

Основным рекомендуемым дизайном прогностических исследований является когортное исследование, которое может быть проспективным или ретроспективным (проспективным по ретроспективно собранным (архивным) данным). Предпочтительным считается проспективный сбор данных, поскольку обеспечивает полный контроль над определением всех потенциальных предикторов и исходов, а также позволяет применять наиболее точные методы их измерения.

В научной литературе и методологических руководствах, посвященных когортным исследованиям, обычно подчеркивается их роль в изучении этиологии заболеваний и факторов риска. В таких этиологических проспективных когортных исследованиях участников распределяют в группы в зависимости от их экспозиции эндогенному или экзогенному фактору — например, курящие и некурящие. Основной целью таких исследований является оценка возможной причинно-следственной связи между изучаемым фактором и последующим исходом1. Необходимым условием проведения когортных исследований является отсутствие изучаемого состояния на момент включения в исследование. Ключевым элементом является стратификация участников по признаку экспозиции, что позволяет выделить влияние изучаемого фактора с учетом потенциальных вмешивающихся переменных. Такой дизайн дает возможность сравнивать частоту развития исходов между экспонированной и неэкспонированной группами (рис. 1).

**Figure:**
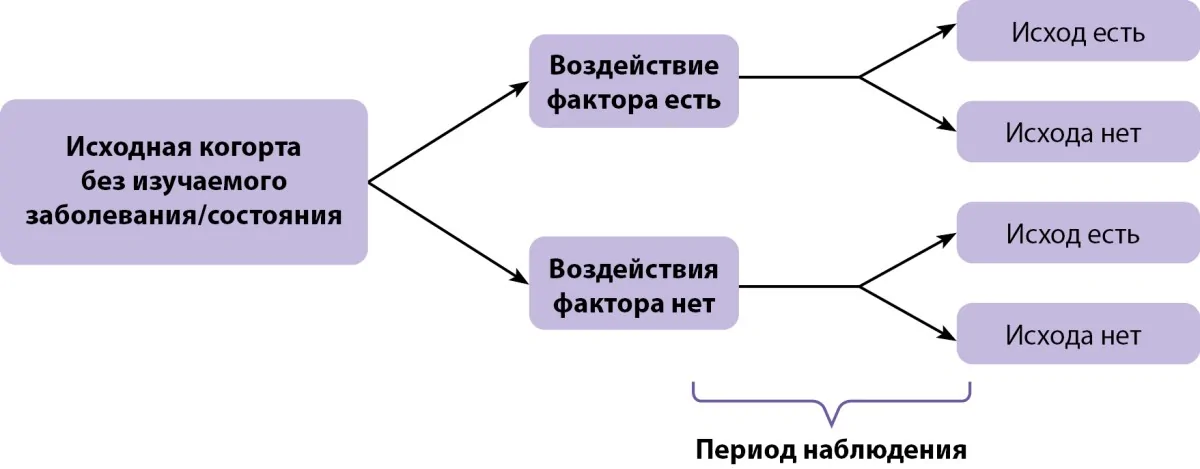
Рисунок 1. Схема этиологического когортного исследования.

Таким образом, когортные исследования этого типа призваны ответить на вопрос: «Может ли фактор А быть причиной заболевания Б?».

Классическим примером этиологического проспективного когортного исследования является исследование British Doctors Study, в котором была доказана связь между курением и заболеваемостью раком легких [2].

В эндокринологии примером этиологического проспективного когортного исследования может быть исследование связи между воздействием внешней радиации и раком щитовидной железы (РЩЖ) в рамках исследования продолжительности жизни (Life Span Study, LSS) [3].

Это исследование является одним из основных долгосрочных проспективных когортных исследований последствий атомных бомбардировок Хиросимы и Нагасаки для здоровья человека. Оно началось в 1950 г. и охватило около 120 000 человек, включая примерно 94 000 подвергшихся и 27 000 не подвергшихся облучению. Одним из долгосрочных последствий радиационного воздействия стало повышение уровня заболеваемости раком щитовидной железы среди населения, подвергшегося воздействию радиации, особенно среди тех, кто получил облучение в детском или подростковом возрасте. Для лиц, подвергшихся воздействию в возрасте 5 лет, при достижении ими 30-летнего возраста избыточный относительный риск на Грей (ERR/Gy)2 составил 4,3 по сравнению с 3,3 для лиц, подвергшихся воздействию в возрасте 10 лет [4].

Минимальный латентный период развития РЩЖ у лиц, подвергшихся радиационному воздействию в детском или подростковом возрасте, составляет приблизительно 5–10 лет. Избыточный риск не достигал максимального значения сразу после воздействия, а постепенно нарастал в течение нескольких десятилетий, как правило, достигая пика заболеваемости через 20–40 лет после облучения. Повышенный риск среди выживших сохранялся на устойчиво высоком уровне даже спустя шесть десятилетий после бомбардировок, что подчеркивает необходимость длительного диспансерного наблюдения за лицами с радиационно-индуцированными злокачественными новообразованиями [5].

В отличие от этиологических когортных исследований, ориентированных на выявление причинно-следственных связей, в прогностических когортных исследованиях ключевая методологическая задача заключается в максимально точном предсказании будущего клинического исхода на основе совокупности исходных переменных (предикторов). Анализ причинности при этом не входит в цели исследования. Основной фокус смещен с объяснения механизмов возникновения исхода на оценку вероятности его наступления, то есть на ответ на вопрос: «Каков наиболее вероятный исход при данных исходных характеристиках пациента?» В связи с этим в прогностических исследованиях применяется особый тип когортного дизайна, не предполагающий исходного распределения участников по группам в зависимости от экспозиции определенному фактору [6][7]. Целью таких исследований является идентификация и количественная оценка исходных характеристик, статистически ассоциированных с последующим развитием конкретных исходов, а не установление причинно-следственных отношений между факторами и исходами.

В прогностических когортных исследованиях исходная когорта формируется из участников, находящихся на одной и той же четко определенной стадии заболевания, либо в момент первого проведения скринингового обследования или начала лечебного вмешательства. Обязательным методологическим условием является отсутствие у всех включенных участников изучаемого клинического исхода на момент начала наблюдения [7]. Включение в когорту осуществляется на основе унифицированных критериев, таких как наличие определенной нозологической формы, пограничного патологического состояния, такого как нарушение толерантности к глюкозе, или факт применения медицинского вмешательства, например, операции по поводу аденомы гипофиза. У всех участников регистрируются исходные и последующие значения широкого спектра потенциальных предикторов, отражающих их клинико-анамнестические, инструментальные и лабораторные характеристики. Дальнейшее наблюдение за когортой осуществляется проспективно с целью регистрации заранее определенных исходов (рис. 2). Формирование групп по наличию или отсутствию изучаемого исхода проводится на этапе статистического анализа, что обеспечивает возможность применения многомерных аналитических методов, включая логистическую регрессию, регрессию Кокса, построение деревьев решений и др.

**Figure:**
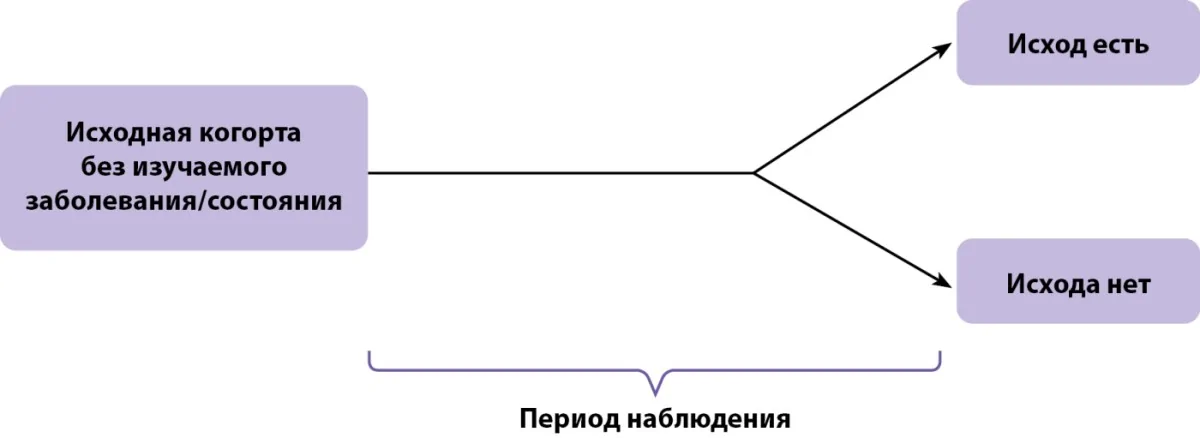
Рисунок 2. Схема прогностического когортного исследования.

В ретроспективных прогностических когортных исследованиях информация о потенциальных предикторах извлекается из архивных источников, содержащих ранее собранные данные о факторах риска, клинических характеристиках пациентов и других значимых параметрах. Аналогично проспективному дизайну, на этапе формирования когорта включает лиц, не имеющих изучаемого исхода. К таким популяциям могут относиться пациенты с впервые установленным диагнозом либо лица, недавно подвергшиеся определенному воздействию, например пациенты, впервые начавшие терапию тиреостатиками по поводу болезни Грейвса.

Использование такого дизайна обеспечивает строгую временную упорядоченность процесса прогнозирования. Начальная точка отсчета времени (например, момент проведения скрининга на сахарный диабет, выполнения медицинского вмешательства или госпитализации) определяется идентично для всех участников, что позволяет задать единую нулевую временную точку (zero time) для предсказания последующих событий [7]. Такая стандартизация исходного состояния и корректный учет предикторов способствуют повышению воспроизводимости и клинической применимости разрабатываемых прогностических моделей.

Благодаря возможности одновременного анализа большого числа переменных и их комбинаций, когортные исследования, не предусматривающие предварительного распределения участников по группам в зависимости от экспозиции изучаемому фактору, представляют собой необходимый и методологически обоснованный дизайн для разработки и валидации прогностических моделей в клинической и эпидемиологической практике.

Поскольку достоверное предсказание будущих событий со стопроцентной точностью принципиально невозможно, прогностические исследования по своей природе носят вероятностный характер и ориентированы на количественную оценку рисков. В отличие от этиологических исследований, которые, как правило, фокусируются на оценке относительного риска3 исхода, ассоциированного с конкретным фактором, прогностические исследования направлены на определение индивидуального абсолютного риска на основе совокупности клинико-лабораторных и анамнестических характеристик пациента [8]. С практической точки зрения именно абсолютный риск — то есть вероятность конкретного клинического исхода в течение определенного периода — является наиболее информативным и ценным для врача и пациента показателем.

В эндокринологии для оценки абсолютного риска применяется множество калькуляторов, номограмм и систем стратификации риска. К ним относятся разработанный Американской диабетической ассоциацией интерактивный онлайн-калькулятор риска СД2 у лиц без сахарного диабета (СД) [9], номограмма для прогнозирования риска гестационного сахарного диабета (ГСД) у женщин с синдромом поликистозных яичников [10], система стратификации риска рака щитовидной железы TI-RADS (Thyroid Imaging Reporting and Data System) на основе ультразвукового исследования [11], калькулятор 10-летнего риска остеопоротических переломов бедра, позвоночника, проксимального отдела плечевой кости или предплечья у лиц в возрасте от 40 до 90 лет (FRAX) [12] и др.

Любая прогностическая модель подлежит обязательной проверке ее прогностических свойств, то есть валидации. Цель валидации заключается в оценке точности и устойчивости модели либо на новых данных той же выборки, не использованных при ее построении (внутренняя валидация), либо на независимых данных, полученных из других источников (внешняя валидация). В условиях ограниченного объема данных вместо классической внутренней валидации применяются методы имитации повторной выборки, такие как кросс-валидация, скользящий экзамен и аналогичные процедуры, что позволяет снизить риск переобучения и повысить надежность прогностических моделей.

## ПРИМЕРЫ ПРИМЕНЕНИЯ ПРОГНОСТИЧЕСКИХ КОГОРТНЫХ ИССЛЕДОВАНИЙ В ЭНДОКРИНОЛОГИИ

Одним из примеров проспективного прогностического когортного исследования в эндокринологии является британское исследование раннего прогнозирования риска ГСД [13].

Целью исследования была разработка модели прогнозирования риска ГСД на сроке 11–13 недель беременности, основанной исключительно на характеристиках матери и данных акушерского анамнеза. Эта цель согласуется с позицией по ГСД британского Национального института здоровья и качества медицинской помощи (NICE). В отличие от Российских клинических рекомендаций по ГСД [14], предусматривающих универсальный скрининг, т.е. обязательное обследование на ГСД всех беременных между 24-й и 28-й неделями, Руководство NICE по ГСД [15] рекомендует прицельный скрининг, т.е. обследование только тех женщин, которые имеют факторы риска ГСД. Поэтому данная прогностическая модель разрабатывалась для идентификации беременных высокого риска по ГСД с целью модификации их образа жизни, усиленного наблюдения и обеспечения последующей диагностики этого осложнения между 24-й и 28-й неделями беременности.

В качестве прогностических факторов в этом исследовании использовались возраст матери, масса тела, рост, расовое происхождение (этничность), семейный анамнез сахарного диабета, использование препаратов для индукции овуляции, масса тела предыдущего новорожденного и наличие ГСД в анамнезе.

В начальную когорту, сформированную в период с 2006 по 2013 гг., вошло 75 161 женщин с одноплодной беременностью. Критериями исключения были диагностированный до беременности сахарный диабет 1-го или 2-го типа, а также случаи беременности, завершившейся прерыванием, выкидышем или родами до 30-й недели. Из 75 161 включенных в когорту женщин у 1827 (2,4%) впоследствии был диагностирован ГСД, в то время как у 73 334 беременных диабет не был зарегистрирован.

После завершения наблюдения был проведен сравнительный анализ включенных в модель предикторов у женщин с диагностированными ГСД и у пациенток без осложнений беременности. На основе проведенного анализа данных с использованием логистической регрессии была создана прогностическая модель, демонстрирующая частоту выявления ГСД (чувствительность) на уровне 83,2% при частоте ложноположительных результатов, составляющей 40%. Модель также продемонстрировала высокую дискриминационную способность: площадь под ROC-кривой (AUC) составила 0,823 (95% доверительный интервал (ДИ) 0,820–0,826). Это означает, что при случайном выборе пары беременных — с ГСД и без него — модель правильно ранжировала риск в 82,3% случаев.

Таким образом, результаты этого исследования свидетельствуют о том, что скрининг ГСД в первом триместре может быть эффективно проведен с использованием неинвазивных и доступных в рамках рутинной практики материнских данных. Это создает возможность для ранней стратификации риска и организации целенаправленного наблюдения за состоянием беременных женщин.

Высокая частота выявления (83,2%) указывает на то, что значительное большинство женщин, у которых позднее разовьется ГСД, будет идентифицировано, тогда как уровень ложноположительных результатов (40 %) — хотя и существенный — может быть приемлемым в контексте скрининга при условии последующего прохождения диагностического обследования.

Это исследование является наглядной иллюстрацией выбора соотношения чувствительности и специфичности прогностической модели в программах раннего выявления заболеваний. Как известно, соотношение между чувствительностью и специфичностью тестов задается в зависимости от их цели (диагностической или скрининговой) и конкретной решаемой задачи – с учетом «цены» ошибок I и II рода (ложноположительных и ложноотрицательных результатов) [16].

В этом исследовании в контексте скрининга ГСД приоритет был отдан высокой чувствительности прогностической модели. Это связано с рисками не выявленного ГСД, включающими преэклампсию, преждевременные роды, инфекции мочевыводящих путей, послеродовое кровотечение и кесарево сечение вследствие крупного плода. В отдаленной перспективе у женщин возрастает вероятность развития СД2, метаболического синдрома и сердечно-сосудистых заболеваний. Для плода отсутствие своевременной диагностики ГСД связано с риском макросомии (масса >4 кг), гипогликемии, гипербилирубинемии, респираторного дистресс-синдрома и родовых травм. В дальнейшем у таких детей увеличивается вероятность развития ожирения, СД2 и неврологических нарушений [17].

Ложноположительные результаты (в этом случае 40%) считаются допустимыми, поскольку усиленное медицинское наблюдение не предусматривает никаких инвазивных процедур и не наносит вреда здоровым беременным. Психологический дискомфорт от ложной тревоги значительно менее критичен по сравнению с последствиями не выявленного ГСД.

Другим примером прогностического исследования в диабетологии является исследование, в котором изучалась возможность использования трех циркулирующих белков — адреномедуллина, TNFR1 и NT-proBNP — для прогнозирования ухудшения функции почек у пациентов с СД2 [18]. Исследование показало, что повышенные исходные уровни этих биомаркеров независимо ассоциируются с более быстрым снижением скорости клубочковой фильтрации и высоким риском тяжплой почечной недостаточности. Эти ассоциации сохранялись после поправки на традиционные клинико-лабораторные факторы, такие как возраст, артериальное давление и креатинин. Таким образом, использование этих биомаркеров, особенно TNFR1, может повысить точность стратификации риска у больных диабетом и способствовать более раннему целенаправленному вмешательству.

В области остеопороза крупное проспективное исследование у женщин в постменопаузе [19] позволило выявить ключевые предикторы риска остеопоротических переломов. Наиболее сильным фактором выступала низкая минеральная плотность кости, однако независимое прогностическое значение имели также наличие переломов в анамнезе и повышенные уровни маркеров костного обмена. Эти результаты подчеркивают многокомпонентный характер прогноза: ни один показатель, включая минеральную плотность, не способен в полной мере объяснить индивидуальный риск, что указывает на необходимость многофакторных моделей с использованием нескольких независимых предикторов.

Примером прогностического исследования в репродуктивной эндокринологии является проспективное когортное исследование [20], посвященное прогнозированию результатов программ экстракорпорального оплодотворения (ЭКО) у женщин с исходно повышенным уровнем фолликулостимулирующего гормона (ФСГ≥12 МЕ/л), которые традиционно рассматриваются как пациентки со сниженным овариальным резервом. Было показано, что хотя высокий ФСГ в целом связан с меньшими шансами на беременность и роды, возраст и количество полученных ооцитов оказываются независимыми и более сильными предикторами успешного исхода.

Прогностическое исследование выживаемости у больных с распространенным адренокортикальным раком (АКР) III–IV стадий [21] продемонстрировало, что модифицированная система стадирования ENSAT (mENSAT), учитывающая размер опухоли, поражение лимфатических узлов и наличие отдаленных метастазов, обладает наибольшей прогностической ценностью в отношении выживаемости. Шкала GRAS, включающая степень злокачественности опухоли, радикальность ее удаления, возраст и наличие симптомов, также продемонстрировала высокую эффективность для стратификации пациентов по уровням риска. Использование этих инструментов позволяет более точно оценивать прогноз и оптимизировать тактику лечения при данном редком агрессивном новообразовании надпочечников.

Во всех этих исследованиях исходная когорта формировалась на основе унифицированных критериев включения. У всех участников в качестве потенциальных предикторов измерялись показатели, характеризующие их клинико-анамнестический профиль в соответствии с целями исследования. В ходе проспективного наблюдения регистрировались случаи развития изучаемого исхода, после чего, по завершении периода наблюдения, когорта разделялась на группы в зависимости от наличия либо отсутствия данного исхода.

Совокупность приведенных примеров иллюстрирует широкие возможности применения такого дизайна в прогностическом моделировании. Такой подход способствует индивидуализированной стратификации риска и поддержке принятия клинических решений в эндокринологической практике.

Когортные исследования с дизайном прогностического исследования также применяются в крупных популяционных исследованиях, направленных на изучения множества факторов риска. Такой дизайн имело знаменитое Фремингемское исследование (Framingham Heart Study), долгосрочное эпидемиологическое исследование, начатое в 1948 г. и продолжающееся до настоящего времени. Оно сыграло ключевую роль в формировании современного представления о влиянии эндокринной патологии на развитие атеросклеротических сердечно-сосудистых заболеваний. В рамках этого проекта было убедительно показано, что сахарный диабет является независимым фактором риска ишемической болезни сердца, сердечной недостаточности и заболеваний периферических артерий [22], выявлена связь метаболического синдрома с сердечно-сосудистым риском [23], а также установлена роль нарушений функции щитовидной железы, включая субклинические формы, в повышенной сердечно-сосудистой смертности и риске ишемических событий [24].

## МЕЖДУНАРОДНЫЕ СТАНДАРТЫ

В соответствии с рекомендациями Международного комитета редакторов медицинских журналов (ICMJE)4, при подготовке научных публикаций авторам следует ориентироваться на руководства, представленные на международной платформе EQUATOR Network [25]. В отношении наблюдательных и прогностических исследований ключевыми документами являются руководства STROBE [26] и TRIPOD [27].

Руководство STROBE предназначено для обеспечения полноты и прозрачности описания наблюдательных исследований, включая когортные, тогда как руководство TRIPOD направлено на стандартизацию отчетности о разработке, валидации и применении прогностических моделей. Подчеркивается, что использование отдельных предикторов, как правило, не позволяет достичь надежной оценки диагностических или прогностических вероятностей и рисков. Поэтому практически во всех областях медицины широкое распространение получили многофакторные модели, которые могут быть диагностическими (в одномоментных (поперечных) исследованиях) и прогностическими (в динамических (продольных) исследованиях).

Аналогично другим рекомендациям платформы EQUATOR Network, руководства STROBE и TRIPOD включают контрольные перечни (checklists), охватывающие все ключевые аспекты исследования — от его дизайна до представления результатов. По каждому пункту приведены пояснения и примеры, что способствует стандартизации структуры научных публикаций и снижает риск упущения значимых элементов при подготовке рукописей статей. Во многих журналах предоставление заполненных контрольных перечней с указанием страниц, на которых представлена соответствующая информация, являются обязательным требованием к направляемым в редакцию рукописям.

Систематическое использование рекомендаций STROBE и TRIPOD вносит вклад в повышение воспроизводимости и прозрачности результатов научных работ, облегчает их критическую оценку рецензентами и редакторами и способствует более эффективной интеграции данных наблюдательных и прогностических исследований в клиническую практику.

## ОШИБКИ ДИЗАЙНА И АНАЛИЗА ДАННЫХ

Одной из наиболее распространенных и концептуально серьезных ошибок в дизайне прогностических исследований является нарушение принципа временной последовательности между измерением потенциального предиктора и наступлением изучаемого исхода. Эта ошибка возникает, когда исследователи определяют значения потенциально прогностических, динамически изменяющихся во времени показателей после того, как прогнозируемое событие уже произошло, или одновременно с ним. Подобная практика подрывает фундаментальную цель прогностических исследований, заключающуюся в предсказании будущих событий, а не в объяснении уже свершившихся.

Наиболее часто нарушение временной последовательности наблюдается в исследованиях, где формирование групп осуществляется на основании наличия или отсутствия изучаемой патологии. Представим гипотетическое исследование, посвященное прогнозированию почечной недостаточности при диабетической нефропатии. После разделения участников по исходу в этих группах измеряются потенциальные предикторы, которые могут изменяться в период между исходной точкой и событием, такие как биомаркеры или физиологические параметры. Такой подход противоречит базовым принципам прогностического исследования, требующим, чтобы прогнозируемый исход отсутствовал у всех участников на момент включения в исследование. В частности, в исследованиях, посвященных прогнозированию остеопоротических переломов, потенциальные предикторы измеряются задолго до развития переломов, как описано выше [19].

Включение в модель показателей, которые изменяются между исходной точкой и наступлением события и измеряются уже после его реализации, представляет собой серьёзное методологическое нарушение. В подобных случаях утрачивается ключевой временной аспект, определяющий последовательность событий и направленность ассоциации между предполагаемым фактором риска (предиктором) и исходом. В результате становится невозможно установить, что предшествовало чему: изменение исследуемого биомаркера или само развитие клинического события.

Например, в прогностическом исследовании почечной недостаточности при диабетической нефропатии формирование групп осуществляется на основе уже установленного диагноза почечной недостаточности, а концентрация предполагаемого биомаркера определяется одновременно с постановкой диагноза или позже. В этом случае возникает принципиальный методологический вопрос: отражает ли зафиксированное значение биомаркера его исходный уровень, предшествующий развитию почечной недостаточности, или же оно представляет собой вторичное проявление уже сформировавшегося патологического процесса? Если изменение показателя происходит в ответ на само заболевание, он не может рассматриваться как истинный предиктор; его значение ограничивается диагностической, а не прогностической применимостью.

Подобные методологические нарушения существенно снижают достоверность и практическую значимость полученных результатов, лишая выявленные маркеры прогностической ценности и делая их непригодными для индивидуальной оценки риска или разработки профилактических стратегий. Для обеспечения внутренней валидности и интерпретируемости выводов в прогностических исследованиях должен строго соблюдаться принцип временной последовательности, обеспечивая измерение потенциальных предикторов до наступления исследуемого исхода в рамках надлежащим образом спланированных проспективных когортных исследований.

Второй распространенной методологической проблемой подобных исследований является чрезвычайно низкое качество анализа данных [28].

В прогностических исследованиях сложные многомерные аналитические методы нередко применяются при отсутствии у авторов достаточной подготовки и необходимых базовых статистических знаний. Это приводит к некорректным результатам и выводам, искаженной интерпретации данных, снижению достоверности полученных результатов и ухудшению воспроизводимости исследований. Подобная практика указывает на методологические пробелы в организации и проведении работ, когда использование продвинутых аналитических подходов осуществляется без должного понимания принципов статистического анализа.

Основная причина такой ситуации заключается в стремлении исследователей применять «модные» или технологически сложные методы без глубокого осознания их концептуальных предпосылок и ограничений. Часто мотивацией становится желание повысить публикационную активность или следование примеру авторов высокорейтинговых публикаций. Однако использование современных статистических программ без понимания теоретической основы биостатистики создает лишь иллюзию научной строгости, которая в действительности может быть ложной. Важно, чтобы исследователи либо повышали собственную статистическую компетентность, либо привлекали к работе квалифицированных специалистов по анализу данных.

Существенной и распространенной ошибкой при выполнении многомерного прогнозирования является неверная подготовка данных (без учета времени наступления исхода и сроков наблюдения пациентов) и соответствующий неправильный выбор метода математического моделирования. Например, после нейрохирургического лечения аденомы гипофиза у пациентов возможны рецидивы, которые регистрируются в разные сроки послеоперационного наблюдения — от нескольких месяцев до десятков лет. Тогда если у пациента нет рецидива на сроке 3 года, это не значит, что он не возникнет на сроке 5 лет, и считать этот конкретный случай негативным при построении прогностической модели нет оснований. Правильным подходом в таком случае является либо фиксация определенного горизонта прогнозирования, как, например, в исследовании Nadezhdina EY и др. [29], либо учет точного времени возникновения исходов и применение соответствующих математических моделей (например, регрессии пропорциональных рисков Кокса).

Еще одной типичной ошибкой при проведении прогностических исследований является использование отдельных предикторов и ROC-анализа в качестве самостоятельного инструмента прогнозирования вместо построения полноценной прогностической модели. Как правило, в таких случаях анализируется бинарный клинический исход и оценивается прогностическая ценность количественных потенциальных предикторов — например, концентраций биомаркеров или ультразвуковых параметров. По полученным данным строятся ROC-кривые, вычисляется площадь под кривой и определяется оптимальное пороговое значение, после чего результат ошибочно представляется как прогностическая модель. Между тем, в соответствии с руководством TRIPOD, прогностическая модель определяется как комбинация нескольких предикторов, объединённых с целью оценки вероятности наступления конкретного исхода или события в определённый момент времени в будущем.

Для объединения предикторов в рамках прогностического моделирования применяются разнообразные статистические методы, которые условно можно разделить на классические подходы и современные методы машинного обучения. Среди традиционных инструментов наибольшее распространение получили регрессионные модели: линейная регрессия используется для анализа непрерывных исходов, тогда как логистическая регрессия применяется при бинарных исходах. При исследовании времени до наступления событий используется модель пропорциональных рисков Кокса. Выбор конкретного метода определяется типом данных, целями исследования и характеристиками изучаемой выборки. В клинических исследованиях предпочтение, как правило, отдается регрессионным моделям благодаря их интерпретируемости и возможности получения клинически осмысленных результатов. В противоположность этому, методы машинного обучения нередко воспринимаются как менее прозрачные и функционирующие по принципу «черного ящика».

В прогностических исследованиях ROC-анализ играет ключевую роль при оценке качества построенной модели, сравнении альтернативных моделей и выборе оптимальных пороговых значений прогнозируемой вероятности для бинарной классификации. В таких исследованиях ROC-кривая представляет собой графическое отображение соотношения чувствительности и специфичности при различных значениях прогнозируемой вероятности (непрерывного показателя в диапазоне от 0 до 1). Основными характеристиками эффективности модели являются дискриминация (discrimination) и калибровка (calibration).

Дискриминационная способность модели отражает её способность различать пациентов с наличием и отсутствием целевого исхода. Для её количественной оценки вычисляют площадь под ROC-кривой и статистически проверяют, отличается ли она от значения 0,5, соответствующего случайной классификации. Калибровочная кривая демонстрирует согласованность между предсказанными вероятностями и наблюдаемыми частотами исходов. Оптимальным способом ее представления считается калибровочный график, на котором значения предсказанной вероятности откладываются по оси X, а наблюдаемые относительные частоты исхода — по оси Y.

Кроме того, для выбранных пороговых значений вероятности необходимо рассчитывать классификационные показатели модели, включая чувствительность, специфичность, прогностическую ценность положительного результата (ПЦПР), прогностическую ценность отрицательного результата (ПЦОР) с возможной поправкой на распространенность исхода и другие метрики. Эти показатели характеризуют эффективность модели при выбранных пороговых значениях вероятности. Следует подчеркнуть, что ограничение оценки только чувствительностью и специфичностью является методологически недостаточным. В клинической практике решения принимаются исходя преимущественно из прогностических ценностей, именно они определяют прикладную значимость модели. При этом нередко наблюдается ситуация, когда модель демонстрирует высокое значение лишь одной из двух прогностических ценностей: высокой ПЦПР — при подтверждении диагноза, или высокой ПЦОР — при его исключении. В таких случаях применение модели оправдано только для соответствующих прогностических сценариев. При оценке всех операционных характеристик необходимо рассчитывать ДИ, а точность модели должна интерпретироваться консервативно — по нижним границам ДИ.

Если же ROC-анализ проводится не для оценки прогностической модели, а для отдельного предиктора, то ROC-кривая строится не по прогнозируемым вероятностям исхода, а по значениям самого количественного показателя (например, концентрации биомаркера). В этом случае ROC-анализ используется для определения порогового значения, которое оптимально разделяет наблюдения с наличием и отсутствием исхода. Однако подобный подход обеспечивает лишь бинарную дискриминацию («исход будет/исхода не будет») и не позволяет вычислить индивидуальную вероятность события, то есть количественный прогноз абсолютного риска. Следовательно, такой анализ не отвечает основным целям прогностического моделирования и может рассматриваться только как этап идентификации потенциальных кандидатов в предикторы, подлежащих последующему включению в многофакторную модель. Кроме того, оценка изолированных предикторов не учитывает влияние возможных вмешивающихся факторов, что может привести к завышенной оценке их прогностической значимости.

Еще одной частой ошибкой является некорректная интерпретация результатов моделирования. Подобно тому, как корреляционные зависимости не свидетельствуют о причинно-следственных связях, прогностические модели также не должны рассматриваться как доказательства причинного влияния предикторов на исход. Их задача — не объяснять механизмы, а обеспечивать статистически обоснованное прогнозирование вероятности события.

В качестве примера ошибок методологии прогностических исследований приводим сравнительно новую, но быстро распространяющуюся разновидность дефектов методологии и анализа данных — фетишизацию методов машинного обучения и искусственного интеллекта. В этом случае эти инструменты воспринимаются как универсальные и самодостаточные «черные ящики», способные в полуавтоматическом режиме извлекать клинически значимую информацию практически из любых массивов данных и обеспечивать высокую точность прогнозов. Такое восприятие приводит к подмене методологического анализа технологическим энтузиазмом, когда внимание исследователей смещается с качества данных, дизайна исследования и клинической интерпретируемости модели на сам факт применения «интеллектуальных» алгоритмов. Например, в исследование Андрейченко А.Е. и др. [30] по прогнозированию госпитализации больных с сахарным диабетом была включена 170 141 запись 23 742 пациентов с СД, а для создания моделей применялась логистическая регрессия, методы градиентного бустинга (LightGBM, XGBoost, CatBoost), методы, основанные на деревьях решений (RandomForest и ExtraTrees), а также алгоритм на основе нейронных сетей (Multi-layer Perceptron). Такой выбор методов выглядит самоцелью — продемонстрировать техническую сложность анализа, а не обеспечить его научную достоверность. Это нагромождение моделей лишь создает иллюзию строгости, не компенсируя критических методологических изъянов.

При этом в список предикторов включены показатели, которые являются симптомами неотложных состояний при СД, требующих госпитализации5, а также количество госпитализаций в течение 12 месяцев. Ключевая методологическая проблема заключается в использовании в качестве предикторов симптомов и признаков, которые сами по себе являются непосредственными показаниями к экстренной госпитализации, то есть частью клинического эпизода, рассматриваемого как изучаемый исход. По сути, модель обучается предсказывать госпитализацию по тем же клиническим признакам, которые врач использует для принятия решения о ней. Это приводит к циркулярной логике: модель «предсказывает» исход, используя его же определители, что делает результаты исследования клинически бесполезными. Включение в модель количества госпитализаций за предыдущий год почти тождественно исходу, а также создает зависимость модели от специфики использования стационарной помощи и организационных особенностей системы здравоохранения, а не от стабильных характеристик пациента.

В результате модель фактически не прогнозирует риск госпитализации, а описывает структуру и частоту уже сложившихся неотложных состояний и врачебных решений о госпитализации.

Следует также отметить, что о количественной оценке риска госпитализации пациентов с СД под влиянием тех или иных факторов в этом исследовании даже не упоминается.

Прогностическая модель в такой популяции должна базироваться на стабильных характеристиках пациента (возраст, длительность СД, осложнения, сопутствующие заболевания, лабораторные тесты, предыдущее использование службы здравоохранения и др.), а не на острых симптомах, являющихся показаниями к госпитализации.

Интересно, что эта же группа авторов опубликовала аналогичное исследование, в котором таким же способом прогнозировалась госпитализация пациентов с артериальной гипертензией [31].

## ЗАКЛЮЧЕНИЕ

Прогностические исследования занимают важное место в построении устойчивой и эффективной клинической практики, которая ориентирована на предоставление качественной медицинской помощи, профилактику болезней и сохранение здоровья.

Учитывая важность надежных оснований для принятия клинических решений в эндокринологической практике, при проведении научных исследований необходимо обеспечить тесное взаимодействие медицинских специалистов с профессионалами в области биостатистического анализа. Внедрение статистического рецензирования в научно-медицинских журналах также являются ключевой мерой, направленной на повышение надежности и научной обоснованности публикуемых работ.

## ДОПОЛНИТЕЛЬНАЯ ИНФОРМАЦИЯ

Источники финансирования. Работа выполнена по инициативе авторов без привлечения финансирования.

Конфликт интересов. Авторы заявили об отсутствии потенциального конфликта интересов.

Участие авторов. Все авторы одобрили финальную версию статьи перед публикацией, выразили согласие нести ответственность за все аспекты работы, подразумевающую надлежащее изучение и решение вопросов, связанных с точностью или добросовестностью любой части работы.

1. Когортные исследования при определенных условиях могут убедительно свидетельствовать в пользу причинно-следственной связи, особенно если соблюдены дополнительные критерии, такие как большой размер эффекта, согласованность с результатами других исследований и биологическое правдоподобие. Но окончательное доказательство причинно-следственной связи обычно требует проведения экспериментальных исследований, таких как рандомизированные контролируемые испытания (РКИ), которые позволяют минимизировать систематические ошибки и контролировать вмешивающиеся факторы. Однако РКИ не могут проводиться для установления этиологии или факторов риска и течения болезней.2. Избыточный относительный риск (ERR) — это мера увеличения риска заболевания (например, рака) по сравнению с базовым риском в неэкспонированной группе. Он рассчитывается как разница между относительным риском и единицей (ERR=RR−1).3. Относительный риск — отношение вероятности наступления исхода в группе лиц, подвергшихся влиянию экзогенного или эндогенного фактора, к вероятности того же исхода в группе лиц, не подвергшихся влиянию этого фактора.4. International Committee of Medical Journal Editors [http://www.icmje.org/]. Recommendations for the Conduct, Reporting, Editing and Publication of Scholarly Work in Medical Journals [ 2024 Dec].5. Бледность кожных покровов, боль в животе, боль за грудиной, венозный застой легких, жажда, крупноочаговые изменения, легочная гипертензия, лихорадка, нарушение зрения, неритмичные тоны сердца, ослабленное дыхание, отек легких, отеки, почечная недостаточность, сердечная недостаточность, сердцебиение, суправентрикулярная тахикардия, расширенная тень средостения, тошнота, фибрилляция предсердий, хрипы в легких, цианоз кожи.
